# Need for Speed: The Importance of Physiological Strain Rates in Determining Myocardial Stiffness

**DOI:** 10.3389/fphys.2021.696694

**Published:** 2021-07-30

**Authors:** Matthew A. Caporizzo, Benjamin L. Prosser

**Affiliations:** Department of Physiology, Perelman School of Medicine, The University of Pennsylvania, Philadelphia, PA, United States

**Keywords:** viscoelasticity, diastole, diastolic dysfunction, HFPEF, myocardial compliance, cardiac mechanics

## Abstract

The heart is viscoelastic, meaning its compliance is inversely proportional to the speed at which it stretches. During diastolic filling, the left ventricle rapidly expands at rates where viscoelastic forces impact ventricular compliance. In heart disease, myocardial viscoelasticity is often increased and can directly impede diastolic filling to reduce cardiac output. Thus, treatments that reduce myocardial viscoelasticity may provide benefit in heart failure, particularly for patients with diastolic heart failure. Yet, many experimental techniques either cannot or do not characterize myocardial viscoelasticity, and our understanding of the molecular regulators of viscoelasticity and its impact on cardiac performance is lacking. Much of this may stem from a reliance on techniques that either do not interrogate viscoelasticity (i.e., use non-physiological rates of strain) or techniques that compromise elements that contribute to viscoelasticity (i.e., skinned or permeabilized muscle preparations that compromise cytoskeletal integrity). Clinically, cardiac viscoelastic characterization is challenging, requiring the addition of strain-rate modulation during invasive hemodynamics. Despite these challenges, data continues to emerge demonstrating a meaningful contribution of viscoelasticity to cardiac physiology and pathology, and thus innovative approaches to characterize viscoelasticity stand to illuminate fundamental properties of myocardial mechanics and facilitate the development of novel therapeutic strategies.

## Introduction

### Cardiac Viscoelasticity in Health and Disease

Reduced cardiac compliance is associated with aging and broadly observed across multiple etiologies of heart failure (Chen et al., 1998; Zile and Brutsaert, 2002; Kass, 2005; Borlaug et al., 2013b). Stiffening arises from remodeling of the extracellular matrix (van Heerebeek et al., 2008; Chris and Jacobs, 2017; Frangogiannis, 2019), changes to the myocyte cytoskeleton (Cooper, 2006; Sequeira et al., 2014; Chen et al., 2018), and modifications to the sarcomere (Miyata et al., 2000; Le Winter and Van Buren, 2002; van der Velden, 2011; Hamdani et al., 2013; Zile et al., 2015). Since myocardium is viscoelastic, its compliance decreases with increasing rate of strain (Rankin et al., 1977; Harris et al., 2002; Wang et al., 2016), and thus proper measurement of myocardial compliance requires physiological loading conditions. Common assessments of myocardial mechanics utilize highly variable loading conditions, resulting in different interpretations about the role of viscoelasticity in cardiac function (Brutsaert et al., 1971; Liu and Wang, 2019). As such, viscoelasticity remains an understudied and poorly understood property of the heart.

The viscoelasticity of the myocardium is an important determinant of cardiac performance in health and disease (Rankin et al., 1977; Wang et al., 2016). Viscous forces influence the rate of myocardial shortening (de Tombe and ter Keurs, 1992), and as the heart fills with blood in diastole, the stretching of the left ventricle against diastolic blood pressure invokes considerable viscoelastic forces (Hess et al., 1979; Caporizzo et al., 2020). This viscoelasticity arises from the slip of cytoskeletal and extracellular cross links that dissipate energy (Nam et al., 2016), and the force reached prior to bond slip, and therefore the stiffness of myocardium, depends strongly on the rate of stretching (Stroud et al., 2002; Linke and Leake, 2004; Nishimura et al., 2006; Caporizzo et al., 2020). Consequently, stressors such as exercise, which increase heart rate and accelerate diastolic filling, amplify viscoelastic forces (Tsutsui et al., 1993; Fraites Thomas et al., 1997; Kass et al., 2004). With viscoelasticity notably greater in heart disease (Harris et al., 2002; Caporizzo et al., 2020), it is not surprising that exercise intolerance is an early symptom for these patients. Still, our understanding of myocardial viscoelasticity and its regulation in the context of stressors and pathophysiology remains incomplete.

### Viscoelasticity and Cardiac Output: Implications for Myocardial Reserve

Controlling myocardial viscoelasticity may be particularly consequential for maintaining myocardial reserve, the ability of the heart to increase its power output in response to increased demand. Viscous forces proportionally increase ventricular stiffness by the rate of ventricular filling. During exercise, reserve capacity is utilized by increasing the heart rate while maintaining the stroke volume (Barmeyer et al., 2009; Borlaug et al., 2013a). As heart rate increases, so does the filling rate (Smiseth, 2018), creating a condition where elevated viscoelasticity may limit filling during exercise.

**FIGURE 1:**
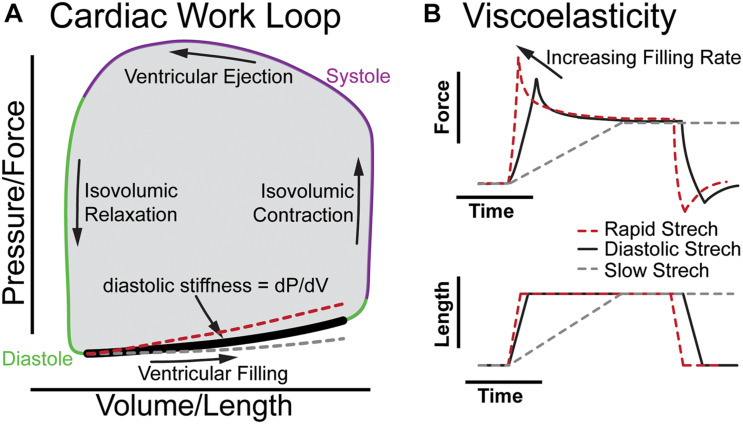
The role of viscoelasticity in determining diastolic compliance. **(A)** Illustration of cardiac work loop with diastolic filling and effective stiffness. The red and gray curves indicate increased or decreased filling rate, respectively. **(B)** The associated force vs. time and length vs. time plots for a diastolic stretch and an isometric hold to reveal viscoelastic relaxation.

Figure 1 illustrates the cardiac work loop and how viscoelasticity changes the shape of the diastolic pressure-volume relationship as filling rate changes. The work of each beat is represented by a plot of ventricular pressure vs. volume forming a “work loop” where the area is the stroke work (Figure 1A). The bottom of the loop represents the pressure and volume increase during diastolic filling, with the slope at any point along the diastolic curve representing the effective stiffness of the left ventricle at that point of diastole (diastolic filling: bold segment Figure 1A). At a resting human heart rate of 60 beats per minute, diastole occurs over about 600 ms divided into less than 100 ms for isovolumic relaxation (downward arrow) and about 500 ms for ventricular filling. The majority of ventricular filling occurs during two intervals of rapid stretching early and late in diastole over which the myocardium strains by 10–20% in about 200 ms separated by a period of diastasis (Smiseth, 2018). Thus, diastole invokes viscous forces from the myocardium and the stiffness of the myocardium depends on the filling rate. In the absence of compensating factors, as the heart rate increases under stress, viscoelastic forces impair filling by increasing the diastolic pressure volume relationship (red dashed line Figure 1A), which would reduce stroke volume, and cardiac output to limit myocardial reserve. In healthy individuals, the beta-adrenergic response may compensate for viscous forces (Fukuda et al., 2005; Falcão-Pires et al., 2011). However, consistent with elevated myocardial viscoelasticity limiting exercise tolerance, patients with diastolic heart failure often exhibit reduced end diastolic volume despite increased diastolic pressure during exercise (Barmeyer et al., 2009; Borlaug et al., 2013a). This underscores the importance of understanding the role of viscoelasticity in cardiac performance.

Figure 1B breaks down the time-dependence of a continuous diastolic stretch to more clearly illustrate the influence of myocardial viscoelasticity on ventricular compliance at different physiological strain rates. This is effectively a portion of the bold segment in 1A split into stress vs. time and volume vs. time. When the length is held constant, the viscoelasticity is evident by the stress relaxation (Figure 1B; Caporizzo et al., 2018). This behavior is not readily appreciated in a cardiac work loop where time is not represented. Yet, the difference between the stiffness at a physiological rate vs a slow rate of strain (dashed gray line) is considerable. Analogously, when the diastolic interval decreases and myocardium is stretched faster (dashed red line) viscoelasticity increases the effective stiffness (Harris et al., 2002; Caporizzo et al., 2018, 2020). On the work loop this behavior appears as a steeper slope of the diastolic pressure–volume curve (red dashed line), which can limit diastolic filling in cases of pathologically increased viscoelasticity (Koide et al., 2000). It should be noted that diastole is a complex multi-component process that is influenced by the lungs, circulatory system, and all four chambers of the heart (Yellin et al., 1992; Kovács et al., 2000), and that while here we use a constant strain-rate to illustrate viscoelastic principles in Figure 1, the left-ventricular myocardium fills at a variable rate in diastole with different phases exhibiting unique mechanical behavior that simple mechanical models alone do not fully capture (Zhang and Kovács, 2008).

### The Cast of Characters: Viscoelasticity in the Myofilament, Myocyte, and Myocardium

Myocardial compliance is regulated at the myofilament, cytoskeletal and extracellular level. At the molecular level tissue viscoelasticity arises from cross-links which break and reform when the material is stretched or compressed. There are numerous such instances of slip bonds in myocardium, such as actomyosin cross-bridges, cell-cell and cell-matrix interactions, and cytoskeletal cross-linking and anchoring complexes. Thus, viscoelasticity is a multi-scale problem with tissue level, myocyte level, and myofilament level contributors.

The myofilaments, which are responsible for the contractile capacity of cardiac tissue, have highly regulated mechanical behavior governed in large part by post-translational modifications and changes in sarcomere stoichiometry (Miyata et al., 2000; Hamdani et al., 2013; LeWinter and Palmer, 2015). The giant protein titin is considered to be a primary determinant of myofilament diastolic tension (Granzier and Irving, 1995) and a significant contributor to myofilament viscoelasticity (Chung et al., 2011). Titin compliance is regulated by phosphorylation, which is commonly altered in disease affecting the diastolic compliance of skinned myocardium (Hamdani et al., 2013; Herwig et al., 2020). Myosin binding protein C acts as a drag, i.e., viscous, element in the sarcomere, bridging the thick and thin filaments in the “C-zones”, and is important for regulating force generation and relaxation (Palmer et al., 2004; Rosas et al., 2015). Myosin activation during systole is the primary determinant of cardiac systolic stiffness, i.e., force generation, although the contribution of cross-bridges to stiffness in the relaxed state remains somewhat uncertain and may depend on conditions (Sequeira et al., 2014). While regulation of myofilament contractile function has an established role in cardiac health and disease, the passive role of myofilament stiffness in regulating diastolic filling is an active area of research.

The non-sarcomeric cytoskeleton consisting of microtubules and desmin intermediate filaments is critical for sarcomere organization (Robison et al., 2016) and regulates myocyte viscoelasticity (Caporizzo et al., 2020). The viscoelastic contribution of microtubules requires their post translational detyrosination which promotes the binding of various microtubule associated proteins that stabilize the microtubule network and promote its cross-linking with the myocyte cytoskeleton and intermediate filament network (Cooper, 2006; Kerr et al., 2015; Chen et al., 2018). Desmin intermediate filaments are elastic elements in the myocyte that wrap around the myofilament Z-disk to maintain sarcomere registry. In heart failure, there is an increased abundance of both detyrosinated microtubules and desmin intermediate filaments (Chen et al., 2018). Together, the microtubule and desmin filament networks form a lattice-like scaffold that contributes to myocyte viscoelasticity facilitates sarcomere maintenance and organization (Caporizzo et al., 2019).

Deposition of extracellular matrix, i.e., fibrosis, is a common feature of dilated and ischemic heart failure in particular (Gulati et al., 2013; Perestrelo et al., 2021). When the ventricular wall is weakened by high filling pressures or ischemic insult, stiffening through fibrosis is necessary to prevent diastolic overstretching or even wall rupture (Nagai et al., 2014). Fibrosis can dramatically stiffen myocardium and is largely thought of as an irreversible feature of disease, as it is resistant to remodeling (Webber et al., 2020). Aside from fibrosis, the cardiac extracellular matrix is rich in viscoelastic proteoglycans that are linked to hypertrophic signaling and overexpressed in multiple etiologies of heart failure (Christensen et al., 2019). The basement membrane consists of type four collagens, laminins, nidogen and other specific ECM proteins which serve as the interface for cell surface receptors (Perestrelo et al., 2021). The overall composition of the ECM is altered in disease independent of fibrosis, but the influence composition has on myocardial viscoelasticity or myocyte remodeling remains unclear.

### In a Non-linear Viscoelastic Myocardium–What Is the Right Mechanical Test?

Like most biological material, myocardium is a non-linear viscoelastic material (Humphrey et al., 1990; Harris et al., 2002; Linke and Leake, 2004), meaning that its stiffness depends on both the rate and magnitude by which it is stretched or compressed. In an intact heart, the sarcomere length typically ranges from about 1.8 μm in systole to 2.1 μm in diastole (Sonnenblick et al., 1967). Depending on the magnitude of strain, structural proteins such as microtubules, titin, and collagen become aligned and maximally extended, which sharply increases their contribution to stiffness. Filamentous elements in the myocardium typically run parallel to the contractile axis of the cardiomyocytes, which form an anisotropic cross-hatched pattern in the ventricular free wall. When sarcomere length is extended beyond 2.1 um, the non-linear stiffening associated with fiber alignment of titin and collagen becomes the dominant factor determining myocardial stiffness (Linke and Leake, 2004; Zile et al., 2015; Caporizzo et al., 2020). This length-dependent stiffening is important to prevent the heart from becoming overstretched or injured but adds a degree of complexity in designing experiments to measure physiologically relevant viscoelastic properties.

In addition to non-linear mechanical properties, the heart expands during diastole, meaning that the myocytes are stretched along both their length and width which leads to compression along their axis normal to both the contractile axis and ventricular wall. Tensile tests only stretch the material in 1D, while compressive tests do not typically stretch sarcomeres, as compression is commonly applied orthogonal to the contractile axis. 2D stretching assays are more physiologically relevant but are highly specialized and difficult to employ. The easiest physiological approximation of diastolic compliance likely comes from stretching myocytes or myocardium in 1D with 10–20% strain in about 100–200 ms along the contractile axis. At these rates myocardial stiffness is increased by about 30–50% by viscoelastic forces (Caporizzo et al., 2020).

The complexity of a non-linear, anisotropic, viscoelastic material makes it difficult to extrapolate physiological implications from a single measurement technique. Yet the use of orthogonal approaches, when combined with varying strain and loading rates and modeling, enables the contributions of sarcomeres, microtubules, and extracellular matrix to be isolated.

### The One Dimensional: Force-Length Relationship

One dimensional mechanical tests are the most common form of measuring myocardial mechanics enabling assessment of both systolic and diastolic properties. Isometric force generation is a typical method to determine the contractile capacity of the heart, conducted by holding the muscle at constant length and introducing calcium to demembranated myocardium (Sequeira et al., 2014) or by electrically stimulating intact myocardium. Similarly, the elasticity of relaxed myocytes or myocardium can be determined by measuring the tension held at various sarcomere lengths. While “resting tension” is an important metric of diastolic stiffness, it only captures elasticity and is effectively a minimum value of stiffness. Tensile tests determine the stress-strain response and can be conducted at physiological strain rates where viscoelasticity is observed (Linke and Fernandez, 2003; Caporizzo et al., 2018). In these experiments, precise length control and force-feedback is possible and has been used to mimic the cardiac cycle to generate work loops in beating myocytes and myocardium (Helmes et al., 2016). These experiments clearly demonstrate that both active and passive myocardial mechanics depend on both the length (Chung, 2020) and the rate of shortening or lengthening (Sonnenblick, 1962; Brutsaert et al., 1971; Chung et al., 2017).

### Force Spectroscopy

Scanning probe-based force spectroscopy employs micro and nanoscale cantilevers (Lieber et al., 2004; Lyon et al., 2009), fluid jets (Swiatlowska et al., 2020), and suction pipettes, to deform myocytes and myocardium to determine viscoelastic properties. Scanning probe microscopy offers the advantage of high spatial resolution that can resolve local stiffness differences between T-tubules, intercalated disks, Z-disks, and M-lines. Scanning nanojet microscopy has been employed for example to demonstrate that detyrosinated microtubules contribute significantly to both the structure and stiffness of the cardiomyocyte cortex at the costamere (Swiatlowska et al., 2020). Determining viscoelasticity requires making measurements at different strain rates or frequencies and modeling the rate-dependence of the modulus. This can be done by either indenting at different speeds (Caporizzo et al., 2015) or by pressing on the material and then oscillating the probe in a low-amplitude frequency sweep to determine the elastic and viscous components of the stiffness (Grant et al., 2012). These experiments find that cardiomyocytes are best modeled by a standard linear solid with an additional parallel viscous element (Robison et al., 2016; Caporizzo et al., 2018). Various groups have successfully observed increased viscoelasticity of cardiomyocytes in heart disease with atomic force microscopy (Nishimura et al., 2006; Chen et al., 2018). However, it is challenging to extrapolate compressive or cortical stiffness, where myocytes are compressed orthogonal to their contractile axis, to physiological tissue level mechanics, where myocytes are stretched along their contractile axis. These techniques also rely on smaller strains than are typically seen in the cardiac cycle, where larger viscoelastic contributions are observed. Nevertheless, it has unrivaled mechanical resolution, making it a go-to technique for determining local or element-specific stiffness of intact samples.

### *In vivo* Assessment of Viscoelasticity

Echocardiography is routinely employed to measure chamber dynamics in patients as it allows for an easy and non-invasive assessment of cardiac systolic and diastolic function (Hildick-Smith, 2001). Echocardiography can be used to characterize diastolic dysfunction, but determining the underlying cause from imaging alone can be difficult (Nagueh, 2020). Quantitative determination of ventricular stiffness *in vivo* requires the simultaneous collection of chamber pressure and volume, as achieved using conductance catheter hemodynamic assesment (Ogilvie et al., 2020). Typically, during these procedures patients are anesthetized and their heart rate is constant. Thus, altering filling rate to assess viscoelasticity is impractical. Lifting patients’ feet to transiently increase venous return is one way to accelerate LV filling (Elwan et al., 2018) and could enable an estimate of *in vivo* viscoelasticity. While crude, this type of procedure can provide insight into the rate-dependent mechanical response, and may provide useful information about a patient’s exercise capacity and the severity of their underlying disease.

### Practical Considerations

**FIGURE 2:**
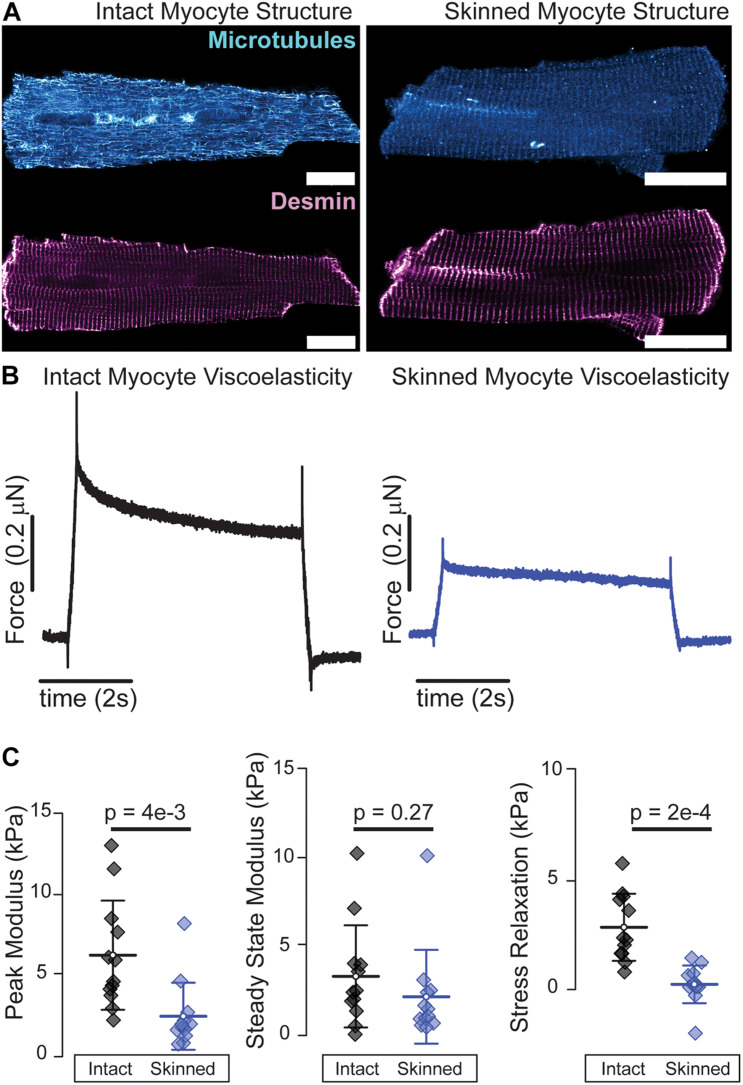
Skinned cardiomyocytes exhibit a loss of microtubules and reduced viscoelasticity. **(A)** Representative fluorescence images of microtubules and desmin intermediate filaments in an intact (left) and skinned (0.1% Triton X-100 30 min at room temperature) (right) cardiomyocyte. **(B)** Average viscoelasticity of intact cardiomyocytes (left) and skinned cardiomyocytes (right). **(C)** Average data for peak, steady-state and stress relaxation, mean line with SD whiskers; *p*-values determined from paired-sample *t*-test. *N* = 12 rates and *n* = 12 myocytes for each. Viscoelastic test is a 15% length step in 200 ms. Scale bar = 20 μm.

Many of the aforementioned mechanical interrogations are conducted in reduced preparations of myofibrils, myocytes, and myocardium at either room or physiological temperature. Both temperature (Templeton et al., 1974) and chemical permeabilization, i.e., “skinning,” affect myocyte viscoelasticity (King et al., 2011). Microtubule polymerization dynamics are sensitive to temperature and while microtubules generally remain intact at room temperature, exposure to ice cold solutions as is common in myocardial preparations can depolymerize a significant fraction of microtubules (Fassett et al., 2019). Skinned preparations allow for robust attachment of myocytes via cytotoxic glues that enable repeated stretching beyond sarcomere lengths of 2.1 μm which is difficult to achieve in intact preparations that rely on myocyte-friendly extracellular glues such as Myotak^TM^. Skinning leads to an expansion of the myofilament lattice that reduces diastolic stiffness at physiological (King et al., 2011) and room temperature (Figures 2B,C) which can be restored by adding high molecular weight polymers such as 2–4% dextran to solution (McDonald and Moss, 1995; King et al., 2011). Skinning also removes key viscoelastic elements such as membranes, microtubules (Figure 2A), and cytosolic proteins, which is reflected in the reduced viscoelasticity of skinned preparations as shown by a greater than proportional decrease in the stress relaxation of skinned myocytes, Figure 2C. “Gentler” permeabilization techniques such as alpha toxin and saponin have been employed to better retain membrane-bound proteins than typical ionic surfactants (Kuznetsov et al., 2008), but while these do retain membrane proteins, the loss of soluble cytosolic factors and the breakdown of structural elements such as the microtubules can still compromise viscoelasticity. Thus, while skinned myocytes represent a powerful and informative platform for interrogating myofilament mechanical properties, one needs to keep in mind that such reductionist approaches can significantly reduce the viscoelasticity of the cardiomyocyte.

### Concluding Remarks

Overall, myocytes and myocardium exhibit non-linear viscoelasticity, meaning that ventricular mechanical properties depend on both the amount and speed they stretch. Myocardial viscoelasticity is a determinant of diastolic stiffness and likely influences cardiac exercise capacity. Ventricular viscoelasticity increases in heart failure due to multiscale remodeling of myofilaments, microtubules, and the extracellular matrix, and while much of these changes have been well characterized in end-stage heart failure, their role in the physiology of cardiac performance is an active area of research. Different viscoelastic characterization techniques provide meaningful information about different aspects of cardiac stiffness, but relating viscoelastic parameters to *in vivo* function remains a challenge. Directly assessing myocardial viscoelasticity *in vivo* also presents a challenge as it necessitates modifying venous return during hemodynamic assessment. Nevertheless, emerging research demonstrating a causal role of increased viscoelasticity in the pathology of heart failure motivates the development of new characterization techniques and strategies to manipulate this understudied component of cardiac mechanics.

### Experimental Methods

Cell Stretch: Adult cardiomyocytes were isolated by Langendorff perfusion as described previously (Robison et al., 2016). Myocytes were then placed in culture in a 95/5 incubator at 37°C in M199 media supplemented with 20 mM HEPES pH 7.4, 1× Insulin Selenium and Transferrin (Thermo Scientific 51500056), 1 mg/mL Primocin (Invivogen), and 25 μM Cytochalasin D (Cayman Chemical Company). Following incubation, cells were exchanged into room temperature normal Tyrode’s solution (Robison et al., 2016) containing 30 mM BDM and attached to the Myostretcher Apparatus with Myotak (Ion Optix LLC). Intact myocytes were stretched via a 200 ms ramp and held at length for 5 s as shown in Figure 1B which corresponded to a stretch from an average sarcomere length of 1.9 μm resting to 2.1 μm stretched. Tyrode’s was then exchanged for relaxing buffer (7.8 mM ATP, 20 mM phosphocreatine, 20 mM imidazole, 4 mM EGTA, 12 mM Mg-propionate, and 97.6 mM K-propionate) and allowed to equilibrate for 5 min before relaxing buffer containing 0.1% T × 100 was introduced into the chamber. Myocytes were continuously observed using the eye piece and transmitted light on a Zeiss Axio Observer Z.1 inverted optical microscope with a Zeiss 1.4 NA 63× oil immersion objective for 30 min of demembranation at which point cell contrast was notably reduced but sarcomere length was still clearly visible. An identical stretch (in terms of resting to stretched sarcomere length) was conducted on the skinned myocyte to determine the skinned cell viscoelasticity. Peak, steady state, and stress-relaxation was quantified from the resulting stress-strain plots. Experiments were conducted at a room temperature of ∼22C.

Immunofluorescence: Cardiomyocytes were gravity sedimented in media and resuspended in relaxing buffer containing 0.1% T × 100 for 25 min on a rocker before gravity sedimentation for the final 5 min of skinning. Supernatant was removed and cells were then immediately fixed in chilled methanol (–20°C) for 7 min. Following fixation, cells were exchanged into blocking buffer and incubated with primary antibodies for alpha tubulin (mouse DM1A–Cell Signaling Technology 1:500) and desmin (goat Y-20, Santa Cruz Biotechnology- 1:200) for 48 h. Cells were then rinsed and exchanged into secondaries (Thermo Fisher Scientific, A10037 and Life Technologies, A-21082) for 48 h of incubation. Cells were then pelleted and resuspended in Prolong Diamond antifade (Invitrogen) and mounted between and glass slide and coverslip for imaging.

Confocal imaging was conducted on a Zeiss 980 laser scanning confocal microscope equipped with Airyscan through a 63 × oil 1.4 NA objective lens. Image analysis was performed using Zen Black for Airyscan processing and Image J was used for image preparation.

Unadjusted *p*-values were calculated using paired sample *t*-tests in Origin Lab 2019.

## Data Availability Statement

The raw data supporting the conclusions of this article will be made available by the authors, without undue reservation.

## Ethics Statement

The animal study was reviewed and approved by Institutional Animal Care and Use Committee.

## Author Contributions

MC and BP designed the experiments, analyzed the data, and wrote and edited the manuscript. MC conducted the experiments. Both authors contributed to the article and approved the submitted version.

## Conflict of Interest

The authors declare that the research was conducted in the absence of any commercial or financial relationships that could be construed as a potential conflict of interest.

## Publisher’s Note

All claims expressed in this article are solely those of the authors and do not necessarily represent those of their affiliated organizations, or those of the publisher, the editors and the reviewers. Any product that may be evaluated in this article, or claim that may be made by its manufacturer, is not guaranteed or endorsed by the publisher.
